# Anchoring Vignettes in EQ-5D-5L Questionnaire: Validation of a New Instrument

**DOI:** 10.2174/1874434601711010144

**Published:** 2017-10-31

**Authors:** Danila Azzolina, Clara Minto, Stefania Boschetto, Matteo Martinato, Barbara Bauce, Sabino Iliceto, Dario Gregori

**Affiliations:** 1 Unit of Biostatistics, Epidemiology and Public Health, Department of Cardiac, Thoracic and Vascular Sciences, University of Padova, Padova, Italy; 2 University Hospital, Gastroenterology, Padova, Italy; 3 Cardiology Unit, Department of Cardiac, Thoracic and Vascular Sciences, University of Padova, Padova, Italy

**Keywords:** Anchoring vignettes, EQ-5D, SF-12, Chopit model, Patient reported outcome study, Quality of life, Factor analysis

## Abstract

**Background:**

Health Related Quality of Life (HRQoL) is an indicator of patient's physical, psychological and social life. HRQoL is influenced by experience, beliefs, perceptions and expectations, and measures subjective perspective of the patient himself. EQ-5D-5L and SF-12 questionnaires are validated instruments useful to measure HRQoL, increasingly administered in electronic formats.

**Objective:**

The main purpose is to evaluate the feasibility of anchoring vignettes for the EQ-5D-5L questionnaire, with the aim to improve intergroup comparability of responses among different subjects. A comparison with SF-12 questionnaire is carried out.

**Method:**

This is a cross-sectional study conducted at the ambulatories of cardiology of the University Hospital of Padova, in Italy. Thirty-eight subjects with a diagnosis of cardiovascular disease or at risk of cardiovascular disease were enrolled. A factorial analysis has been performed to assess the convergent validity of EQ-5D-5L questionnaire compared to Sf-12. Moreover, a compound Hierarchical Ordered Probit (Chopit) model has been estimated to evaluate if the questionnaire form affects the subjective evaluation process in order to compare EQ-5D-5L with and without vignettes.

**Results:**

Correlation and factor analysis demonstrate that EQ_5D questionnaire is coherent with SF-12 in paper format. Chopit model estimation shows that questionnaire format does not affect the subjective question interpretation. Moreover, in a parametric model including vignettes, education attainment, disease severity, and gender are predictors of HRQoL status.

**Conclusion:**

The EQ-5D including vignettes in electronic format seems to be a valid tool to measure HRQoL as compared to EQ-5D without vignettes in paper format and to SF-12 questionnaire.

## 1 INTRODUCTION

Health related quality of life (HRQoL) measures represent an important part of assessing the quality of routine care in general practice. They are useful in understanding the patient’s point of view about the disease and the treatment methods applied, and deserve important consideration when comparing different treatments methods and evaluating interventions [1].

Nurses are educated to provide a wide range of components of care and they are aware of the importance of the quality of patients’ lives because nursing is holistically concerned with the whole patient and is a caring practice aimed to health promotion and maintenance or restoration of function [2].

There is a growing evidence indicating that 'quality of life assessment' is particularly relevant to the scope of nursing practice [3] and can be considered as adjuvant to clinical and physiological assessments in many chronic conditions, particularly cancers [4] and cardiovascular diseases [5]. This approach is the 'gold standard' in the evaluation of nursing care, healthcare services and outcome assessment.

Health-related quality of life tools have the potential to identify specific and general health needs which is particularly important in nursing dealing with holistic care: components of HRQoL tools are likely to be associated with specific health care needs and measuring HRQoL may lead to improved quality of care and to improvement of patients’ QoL, but the administration of quality of life tools can also provide a rapid screening in order to identify patients’ health needs.

Nevertheless, one of the main barriers in using HRQoL as an outcome indicator in the comparison of different survey results is the so-called *interpersonal incomparability* [6]. The evaluation and decision-making process that leads a respondent to evaluate and choose among different response categories of survey questions is a quite complex phenomenon. This complexity is the result not only of the individuality, but also of socio-cultural factors: same question can be interpreted in different ways by people belonging to different cultural contexts, but this difference can be detected even among individuals belonging to the same cultural field.

These factors may hamper the comparability of survey research especially if they are related to heterogeneous study populations and if the phenomenon under investigation is somewhat abstract [7].

For these reasons, King and colleagues (2004) introduced the anchoring vignettes, a methodological tool that seeks to correct for the different interpretations that can be given to responses on ordinal scales.

The main research purpose in this article is the validation of a touch-screen format EQ-5D-5L questionnaire with anchoring vignettes, through comparison with SF-12 questionnaire in paper format without vignettes.

Furthermore, we investigated whether the questionnaire form (paper or touch screen format) and/or the presence of the vignettes in EQ-5D-5L affects the interpretation process of questions proposed to respondent.

## 2 MATERIALS AND METHODS

### 2.1 Study Design and Setting

The setting of our research is an observational cross-sectional study, conducted at the ambulatories of cardiology of the University Hospital of Padova, in the period between February and March 2015. Patients who underwent medical examination during the period of data recruitment were enrolled in the study. Subjects involved were both patients with cardiovascular disease and patients at risk of cardiovascular disease.

Criteria for eligibility were the age of consent (over 18 years), the absence of any major cognitive impairment and the Italian language as a mother tongue. Verbal informed consent has been provided to all the subjects involved in the study, after an explanation of the aim of the research.

### 2.2 Vignettes Techniques

The vignettes are fictitious questions concerning persons who live in a situation attributed to the phenomenon under investigation (an example is provided in Box **1**). The vignettes are proposed to the respondent in order to increase or decrease adhesion to the concept measured [6].

Through the comparison between responses given to self-assessment questions and vignettes questions, it is possible to overcome incomparability between responses (given by different subjects to the same question) which affects HRQoL evaluations as well.

From a statistical standpoint, Greene and Hensher (2010) and Wand (2011) introduced and reviewed, respectively, the compound hierarchical ordinal probit (Chopit) model, which can be successfully used to analyze ordinal responses to a questionnaire including vignettes [8].

In literature, several instruments have been provided to measure the HRQoL. Among them, the EQ-5D-5L questionnaire is a standardized instrument applicable to a wide range of health conditions and treatments. EQ-5D-5L provides a simple descriptive profile and a single index value for health status consisting in five dimensions [9]: mobility, self-care, usual activities, pain/discomfort, and anxiety/depression.

The electronic version of these questionnaires have been often used recently since they improve the survey’s effectiveness and efficiency by reducing probability of error in data entry process [10].

Other authors use anchoring vignettes in EQ-5D-5L questionnaire in paper format to correct interpersonal incomparability in HRQoL evaluation [11].

### 2.3 Instruments of Data Collection

**Fig. (1):**
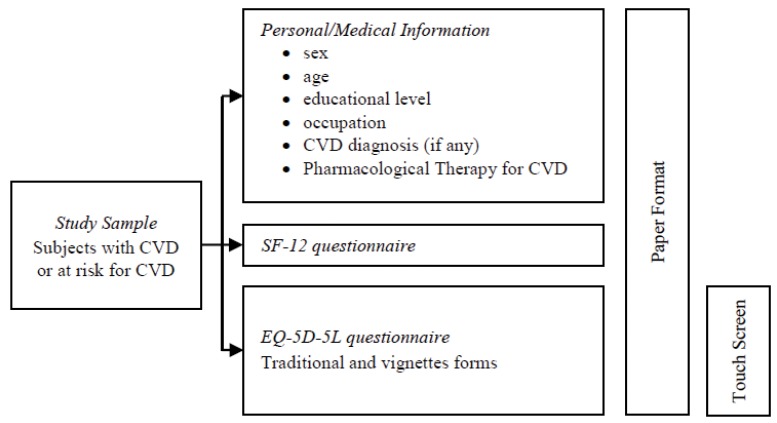
Study design.

Patients completed two different types of questionnaire on quality of life assessment: the EQ-5D-5L questionnaire in traditional and vignettes form, using paper format or touch screen tool, and the SF-12 questionnaire in paper form. Moreover, additional information was collected on age, gender, job, educational level, presence of cardiovascular disease or predisposition, pharmacological therapy and Implantable Cardioverter Defibrillator (ICD) treatment (Fig. **1**).

#### 2.3.1 EQ-5D-5L Questionnaire

Developed by the EuroQol Group, the EQ-5D-5L is the 5-level version of EQ-5D. The questionnaire comprises the same 5 dimensions of previous EQ-5D version (mobility, self-care, usual activities, pain/discomfort, anxiety/ depression), with 5 levels for each dimension: no problems, slight problems, moderate problems, severe problems and extreme problems [12]. EQ-5D has been developed as a simple generic measure to evaluate quality of life [13], and has been validated in several studies on cardiovascular diseases [14-16]. In our study, the same questionnaire has been administered in standard format and in a newest version with anchoring vignettes. Vignettes have been created from the translation of questionnaire proposed by Au and Lorgelly [11].

#### 2.3.2 SF-12 Questionnaire

The SF-12 Questionnaire is an instrument for evaluating health and quality of life perception [17]. It includes 12 questions about 8 different dimensions: physical functioning (PF), role-physical (RP), bodily pain (BP), general health (GH), vitality, social functioning (SF), role emotional (RE), and mental health (MH).

### 2.4 Statistical Analysis

Correlation measures are computed (on the conventional and polycoric correlation matrix) on standardized sum of scores for each dimension of EQ-5D and of SF-12 questionnaire in order to assess the concordance. A factorial analysis on polycoric correlation matrix has been performed to assess the coherence [16] of EQ-5D in vignettes format with SF-12 without vignettes. Results are typically interpreted in terms of the major loadings on each factor and represented either as a table of loadings or graphically, with all the loadings with the absolute value greater than 1 as shown.

A Chopit model has been estimated for the components that may affect the item evaluation process, with the questionnaire form (paper or touch screen) included as an explanatory covariate [18].

The standard parametric analysis presented in this paper is based on the Chopit model [18]. It consists of two sets of response variables, one for self-assessment, and one related to the answers that the interviewee gives to the vignettes. The main difference between the Chopit and probit model lies in the thresholds of the continuous latent variable that defines the response process. Such thresholds are fixed in probit models and varying in Chopit model, depending on the individual characteristics, thus indicating the presence of covariates that affect the subjective evaluation [19].

By using only the component related to self-evaluation, it is not possible to separate the parameters (β) related to self-evaluation component from those (γ) defined on the cut point of latent variable (see Appendix). For this reason, it is important to use the information provided by the vignettes for modeling also this component.

Results of Chopit model, performed on vignettes questionnaire, has been compared with conventional ordinal Probit model performed on data without vignettes.

## 3 RESULTS

Table **1** reports the correlation coefficients between the dimensions included in each questionnaire. These measures of convergent validity between EQ-5D (with vignettes) and SF-12 questionnaire, indicate that anxiety is related with all SF-12 dimensions excluding general mental health (MH), and that physical function (PF) is associated with all EQ-5D dimensions.

**Fig. (2):**
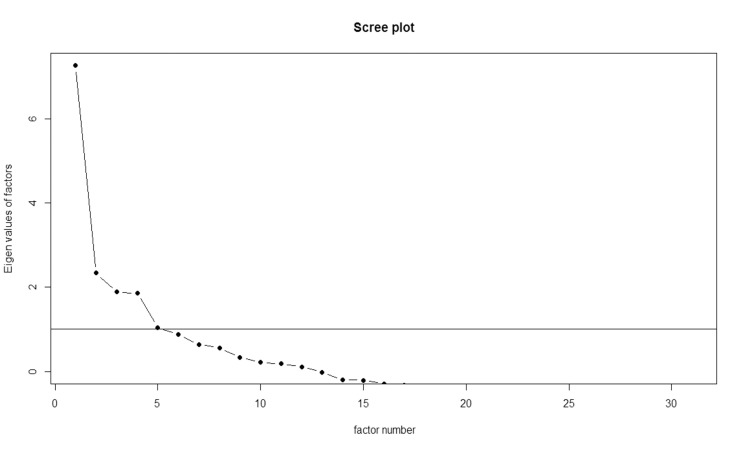
Scree plot of factorial and component analysis on polycoric correlation matrix.

Five latent factors whose eigenvalues are greater than 1 are identified (Fig. **2**).

**Fig. (3):**
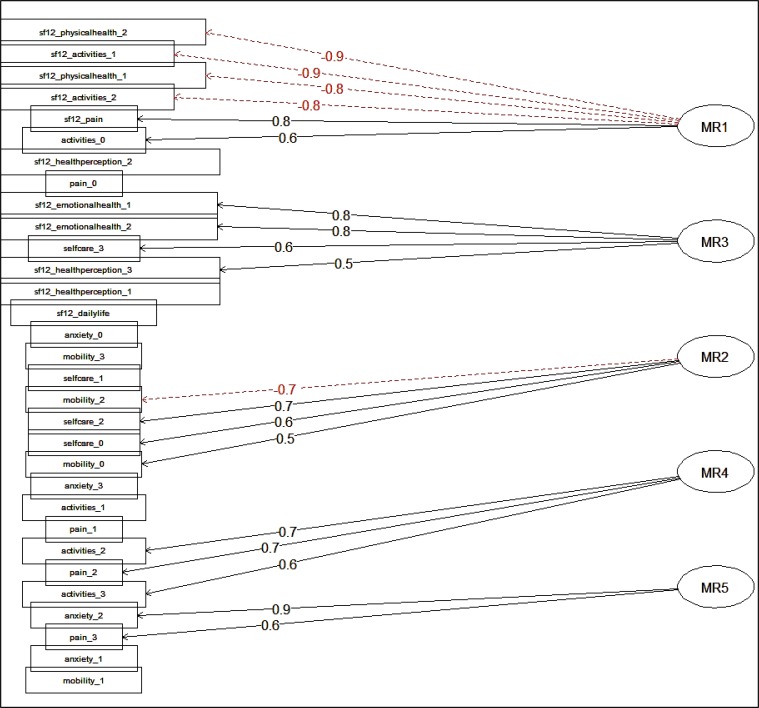
Factor loadings on four latent components(Progressive code numbers at the end of the labels indicate items constituting each section of the questionnaire).

Observing the loadings of variables on latent factors (Fig. **3**) it is possible to assess that, on the first factor, the variables related to physical activity in EQ-5D and SF-12 questionnaire are coherently relevant. The variables with greater loadings on the second factor are related to emotional health and self-care status in both questionnaires. Mobility and selfcare variables are relevant for the third dimension, while on fourth factor are relevant activity and pain component, and the variable more contributing to last factor is the anxiety component. The identified dimensions are coherent with the different aspects of HRQoL, which the questionnaire aims to capture.

A comparison between EQ-5D questionnaire in paper and touch-screen format has been performed evaluating if the questionnaire form affects the subjective evaluation parameters modeled in a Chopit model. The Chopit model has been estimated on each questionnaire dimension.

The questionnaire form is not a significant factor affecting the subjective evaluation, as modeled by *γ* parameters (Tables **2** - **5**).

For each computed model, the questionnaire form, in paper and touch screen format, does not affect the subjective interpretation of the ordinal scale proposed. This result comes from the lack of statistical significance of estimates on threshold defined on the latent variable; the subjective choice is modeled by a normal latent variable on which are defined covariates that may affect subjective evaluation process.

Moreover, the significant estimates (*β*) provided by a Chopit model, including vignettes, are also significant and coherent, in terms of estimated effect, compared to conventional proportional odds model (Tables **2** - **5**), performed on EQ-5D questionnaire without vignettes.

Considering the Chopit model and ordinal Probit model, it seems that the main factor influencing each dimension of the subjective quality of life is represented by the educational attainment. As shown in Tables **2** and **3**, it is statistically significant for mobility and activity dimension (P-value<0.05), indicating lower propensity in people with higher education level to perceive themselves as subjects with problems in mobility (OR=0.33) and activity (OR=0.44).

Gender is related to the perceived quality of life (P-value<0.05), in particular for the anxiety dimension (Table **5**), denoting a greater tendency for females to perceive a poorer quality of life (OR=2.79).

Considering pain dimension, in Table **4**, gender and ICD implantation are associated with subjective pain perception; specifically, women perceive more pain and discomfort (OR=2.58). Moreover, ICD implantation is associated with a higher propensity to pain perception (OR=5.74).

The model has not been estimated for self-care dimension because the majority of individuals in the sample (33 subject) confirmed not to have any problems in washing or dressing themselves, indicating that almost all patients are autonomous in the management of their daily chores.

## 4 DISCUSSION

The aim of the study is the assessment of the coherence between EQ-5D with SF-12 paper questionnaire, a validated tool useful to measure HRQoL outcome. The assessment of convergent validity between EQ-5D and SF-12 indicates that comparable dimensions are more related (*i.e.* Activity versus Physical Function, or Pain versus Bodily Pain and Anxiety versus Vitality and Social Functioning). A similar correlation pattern has been reported in Pattanaphesaj *et al.* [20].

Considering the overall score on EQ-5D in vignettes and SF-12, it is possible to consider that there are five latent dimensions (Health and Physical activity, Emotional health, Selfcare, Pain and discomfort, and Anxiety) coherently with EQ-5D questionnaire structure. Also, other studies confirmed the convergent validity of both instruments to measure the HRQoL [21].

Once an overall coherence between different instruments used to measure the same HRQoL outcome has been established, another objective was to evaluate if the touch screen form of the vignettes questionnaire affect the item interpretation process.

EQ-5D vignettes questionnaire, in paper form, has been considered in literature as a validated instrument useful to take into account of heterogeneity in item interpretation process [11]. It has been shown that EQ-5D questionnaire may be subject to DIF [3] (Different Item Functioning), a different item evaluation process across heterogeneous subjects. If DIF is not considered, the conclusions about perceived HRQoL may be misleading especially when heterogeneous groups are analyzed [19].

Chopit model, performed on vignettes questionnaire, leads to consider covariates on Item evaluation process identifying variables that may affect the subjective question interpretation [7], for this reason, the questionnaire form, has been included in the model as explanatory covariate defined on variable’s interpretation performed by respondent.

Concerning the validation of EQ-5D questionnaire with vignettes in touch screen format, the questionnaire form does not affect the interpretation of questions among subjects, confirming that the electronic format is equivalent with correspondent validated paper tool.

Moreover, as the administration of the electronic questionnaire is a widespread practice, many patient reported outcome studies confirm that paper and computer administered questionnaires are equivalent [22].

Considering also the patient’s prospective, an important aspect in PCOR research, in some studies, patients preferred electronic surveys, especially when being assessed for psychological aspects [23, 24]. A further point under consideration is the evaluation of coherence, in terms of results estimation between a Chopit model performed including vignettes in the data, and the results obtained estimating a conventional Ordinal probit model.

The results are very similar in term of effects related to factors affecting the HRQoL in both estimated models. Consistently with existing literature [25], it is possible to confirm that subjects with higher education attainment level achieve lower scores on EQ-5D scale in mobility, activity and anxiety dimensions, thus indicating a better quality of life in these domains.

Considering pain dimension, the factors affecting the HRQoL seem to be ICD implantation and gender. Other studies confirmed that the disease severity is associated with the subjective pain perception: in fact, the presence of previous chronic disease was associated with higher pain and discomfort perception [26, 27]. As shown in other researches, there is a significant relation between gender and anxiety/depression dimension in the EQ-5D questionnaire [21].

However, we acknowledge that further research is needed to generalize the validity of EQ-5D vignettes questionnaire in electronic form, given that we have built our conclusions on the small study sample. In fact, 90% of articles reporting validation of patient centered outcomes had a sample size greater than or equal to 100 [28].

Moreover, when an abstract concept such as HRQoL is considered, it appears pretty obvious that it is necessary to make most of the vignettes, but in our case the questionnaire has been validated using a sample of fairly homogeneous patients. Vignettes questionnaire is useful to correct DIF especially in case of rising heterogeneity of individuals in terms of socio-cultural characteristics [29]. A further research development may be the validation of EQ-5D questionnaire using a larger sample of patients with more heterogeneous features in terms of health, social background and culture.

## CONCLUSION

The EQ-5D in vignettes in electronic format is a tool to measure HRQoL, which seems as valid as other validated questionnaires used to measure the same concept as SF-12 questionnaire.

Moreover, the questionnaire electronic form, seems to be a factor not affecting the subjective item evaluation process.

## Figures and Tables

**Table 1:** Correlation coefficient and 95% CI between EQ-5D and SF-12 dimensions.

	***PF***	***RP***	***BP***	***GH***	***VT***	***SF***	***RE***	***MH***
***Activity***	-0.34 (-0.6; -0.03)	-0.32 (-0.58; 0)	0.2 (-0.12; 0.49)	0.37 (0.05; 0.62)	-0.04 (-0.36; 0.28)	0.32 (0; 0.58)	-0.17 (-0.46; 0.16)	0.4 (0.1; 0.64)
***Pain***	-0.33 (-0.59; -0.02)	-0.38 (-0.63; -0.07)	0.36 (0.04; 0.61)	0.48 (0.19; 0.69)	-0.24 (-0.52; 0.08)	0.3 (-0.03; 0.56)	-0.24 (-0.52; 0.09)	0.13 (-0.2; 0.43)
***Anxiety***	-0.5 (-0.71; -0.21)	-0.39 (-0.63; -0.08)	0.37 (0.06; 0.62)	0.47 (0.17; 0.69)	-0.54 (-0.74; -0.27)	0.55 (0.28; 0.74)	-0.38 (-0.62; -0.07)	0.15 (-0.18; 0.45)
***Selfcare***	-0.32 (-0.58; 0)	-0.27 (-0.54; 0.06)	0.28 (-0.04; 0.55)	0.21 (-0.12; 0.5)	-0.09 (-0.4; 0.24)	0.04 (-0.29; 0.35)	0.02 (-0.3; 0.34)	0.03 (-0.29; 0.35)
***Mobility***	-0.48 (-0.69; -0.19)	-0.32 (-0.58; 0)	0.23 (-0.1; 0.51)	0.3 (-0.02; 0.57)	-0.1 (-0.41; 0.23)	-0.04 (-0.35; 0.29)	-0.14 (-0.44; 0.18)	0.07 (-0.26; 0.38)

PF (physical functioning), RP (role limitation due to physical problems), BP (bodily pain), GH (general health perceptions), SF (social functioning), VT (vitality), RE (role limitations due to emotional problems), MH (general mental health).

**Table 2:** Results of Chopit and Probit model for mobility dimension, Odds Ratio (OR), Standard error (SE).

	***Chopit*** ***OR(SE)***		***p-value***	***Probit*** ***OR(SE)***			***p-value***
***β age***	1.0269	(0.0263)	0.3136	1.018		(0.0227)	0.4252
***β job***	1.1407	(0.6842)	0.8475	0.9215		(0.5793)	0.8877
***β education***	0.3337	(0.4801)	0.0222	0.5512		(0.3731)	0.1104
***β pharm treat.***	4.0792	(0.6886)	0.0412	3.606		(0.5961)	0.0314
***β questionnaire***	0.2505	(0.9124)	0.1292	0.4302		(0.7797)	0.2793
**Thresholds**							
	***Chopit*** ***Coefficients (SE)***		***p-value***		***Probit*** ***Coefficients (SE)***		***p-value***
***γ1***	-1.4893	(1.8027)	0.4087	***τ*_1_**	-1.387	(1.77)	0.4332
***γ1 age***	0.0119	(0.0139)	0.3919	***τ*_2_**	-0.5809	(0.3394)	0.087
***γ1 job***	0.2545	(0.3797)	0.5027	***τ*_3_**	0.5158	(0.4887)	0.2912
***γ1 education***	-0.4816	(0.2903)	0.0971		-		-
***γ1 pharm treat.***	0.1332	(0.3546)	0.7072		-		-
***γ1 questionnaire***	-0.6554	(0.4901)	0.1811		-		-
***γ2***	0.8298	(1.1505)	0.4707		-		-
***γ2 age***	-0.0204	(0.0129)	0.1138		-		-
***γ2 job***	-0.1951	(0.3201)	0.5422		-		-
***γ2 education***	-0.0068	(0.2208)	0.9754		-		-
***γ2 pharm treat.***	-0.0386	(0.3329)	0.9077		-		-
***γ2 questionnaire***	0.7293	(0.4667)	0.1181		-		-
***γ3***	-0.5081	(1.4315)	0.7227		-		-
***γ3 age***	0.0078	(0.0157)	0.6193		-		-
***γ3 job***	0.4675	(0.4727)	0.3227		-		-
***γ3 education***	0.2338	(0.3322)	0.4816		-		-
***γ3 pharm treat.***	0.1697	(0.422)	0.6876		-		-
***γ3 questionnaire***	0.174	(0.5653)	0.7582		-		-

*γ* are parameters defined on thresholds *β* are the parameters estimated in each model *τ* are the thresholds fixed in Ordinal probit model

**Table 3:** Results of Chopit and Probit model for activity dimension, Odds Ratio (OR), Standard error (SE).

	**Chopit** **OR(SE)**		**p-value**	**Probit** **OR(SE)**				**p-value**
**β job**	1.4232	(0.5359)	0.5102	1.339		(0.5346)		0.5854
**β education**	0.4012	(0.3528)	0.0096	0.3695		(0.3618)		0.0059
**β pharm treat.**	1.748	(0.4907)	0.255	1.763		(0.4885)		0.2459
***Thresholds***								
	***Chopit*** ***Coefficients (SE)***		***p-value***	***Probit*** ***Coefficients (SE)***				***p-value***
**γ1**	-2.3607	(1.3186)	0.0734	***τ*_1_**	-3.035		(1.307)	0.0203
**γ1 questionnaire**	-0.2113	(0.222)	0.3412	***τ*_2_**	-2.038		(0.2991)	0
**γ2**	1.9124	(0.6498)	0.0032	***τ*_3_**	-0.807		(0.48)	0.0926
**γ2 questionnaire**	-0.5577	(0.3228)	0.084					-
**γ3**	2.2796	(1.4109)	0.1062	-				-
**γ3 questionnaire**	-0.5324	(0.6726)	0.4286	-				-
**γ4**	1.4936	(2.0695)	0.4705	-				-
**γ4 questionnaire**	1.1001	(1.3664)	0.4208	-				-

*γ* are parameters defined on thresholds *β* are the parameters estimated in each model *τ* are the thresholds fixed in Ordinal probit model

**Table 4:** Results of Chopit and Probit model for pain dimension, Odds Ratio (OR), Standard error (SE).

	**Chopit** **OR(SE)**		**p-value**	**Probit** **OR(SE)**				**p-value**
***β sex***	2.58	(0.4332)	0.0287	2.551		(0.4335)		0.0308
***β age***	1.0092	(0.0173)	0.5949	1.009		(0.0172)		0.5847
***β icd***	5.7431	(0.8869)	0.0487	5.613		(0.8893)		0.0524
***β job***	0.5368	(0.4826)	0.1974	0.5437		(0.4815)		0.2057
***β education***	0.8983	(0.2823)	0.7042	0.8903		(0.2828)		0.6811
***β questionnaire***	1.0456	(0.5868)	0.9394	1.303		(0.5497)		0.6304
***Thresholds***								
	***Chopit*** ***Coefficients (SE)***		***p-value***	***Probit*** ***Coefficients (SE)***				***p-value***
**γ1**	0.8023	(1.4106)	0.5695	***τ*_1_**	0.8105		(1.401)	0.5628
**γ1 questionnaire**	-0.1958	(0.3069)	0.5235	***τ*_2_**	2.352		(0.2066)	0
**γ2**	1.6839	(0.565)	0.0029	***τ*_3_**	3.606		(0.3665)	0
**γ2 questionnaire**	-0.0685	(0.3292)	0.8352					-
**γ3**	1.1052	(0.6642)	0.0961	-				-
**γ3 questionnaire**	0.0424	(0.3344)	0.8991	-				-
**γ4**	2.2448	(1.3221)	0.0895	-				-
**γ4 questionnaire**	-0.4086	(0.5601)	0.4657	-				-

*γ* are parameters defined on thresholds *β* are the parameters estimated in each model *τ* are the thresholds fixed in Ordinal probit model

**Table 5:** Chopit and Probit model for Anxiety dimension.

	**Chopit** **OR(SE)**		**p-value**	**Probit** **OR(SE)**				**p-value**
***β job***	0.7707	(0.4742)	0.5829	0.9166		(0.4563)		0.8486
***β education***	0.594	(0.3788)	0.1691	0.5606		(0.2949)		0.0497
***β sex***	2.7927	(0.4544)	0.0238	2.935		(0.4347)		0.0133
***β age***	1.0047	(0.0146)	0.7475	1.014		(0.0126)		0.2769
***β pharm treat.***	1.4469	(0.426)	0.3859	1.553		(0.4193)		0.2939
***Thresholds***								
	***Chopit*** ***Coefficients (SE)***		***p-value***	***Probit*** ***Coefficients (SE)***				***p-value***
***γ1***	-0.6444	(1.5548)	0.6785	***τ*_1_**	-0.468		(1.432)	0.7438
***γ1 education***	-0.0672	(0.2137)	0.7531	***τ*_2_**	0.7904		(0.2301)	6e-04
***γ1 questionnaire***	0.0534	(0.2882)	0.853	***τ*_3_**	2.718		(0.3674)	0
***γ2***	3.2064	(1.4287)	0.0248					-
***γ2 education***	-0.1128	(0.2388)	0.6366	-				-
***γ2 questionnaire***	-0.885	(0.4375)	0.0431	-				-
***γ3***	-8.0409	(8.7936)	0.3605	-				-
***γ3 education***	2.5704	(2.6896)	0.3392	-				-
***γ3 questionnaire***	1.4856	(1.7219)	0.3882					
***γ4***	8.18	(12.9886)	0.5288					
***γ4 education***	1.5439	(2.4728)	0.5324					
***γ4 questionnaire***	-2.5932	(3.7446)	0.4886					

**Box (1):** Self assessment question and vignettes question for mobility.

**Mobility (Self Assessment Question)**
1. I have no problems in walking about
2. I have slight problem in walking about
3. I have moderate problems in walking about
4. I have severe problems in walking about
5. I'm unable in walking about
**Vignettes Question:**
• Andrea/Giulia walks for one to two kilometers everyday without tiring, but he/she cannot run anymore due to an injured knee.
1. He/She has no problems in walking about
2. He/She has slight problem in walking about
3. He/She has moderate problems in walking about
4. He/She has severe problems in walking about
5. He/She is unable in walking about
• Luca/Chiara has a lot of swelling in his/her legs, and walking around more than 50 m is an effort as his/her legs feel heavy.
1. He/She has no problems in walking about
2. He/She has slight problem in walking about
3. He/She has moderate problems in walking about
4. He/She has severe problems in walking about
5. He/She is unable in walking about
• Marco/Francesca is able to walk distances of up to 200 m without any problems but feels tired after walking 1 km or climbing up more than one flight of stairs.
1. He/She has no problems in walking about
2. He/She has slight problem in walking about
3. He/She has moderate problems in walking about
4. He/She has severe problems in walking about
5. He/She is unable in walking about

## LIST OF ABBREVIATIONS

HRQoL: = (Health Related Quality of Life)
Chopit: = (Compound Hierarchical Ordered Probit)
ICD: = (Implantable Cardioverter Defibrillator)
DIF: = (Differential Item Function)

## APPENDIX

### Model Specification

Chopit model (Compound Hierarchical Ordered Probit) is an extension of the classical ordinal probit to the case where a dataset also includes vignettes. The methodology consists of two sets of response variables, one for self-assessment, and one relating to the answers that the interviewee gives to the vignettes.

In this context, *Y_i_* represents the response of the *i-th* subject given to self-assessment *(for i = 1,…,n)*, and *Z_lj_* is the individual score *(for l = 1,…,L)* attributed to the *j-th* vignette *(for j = 1,…,2J+1)*, taking into account that both the vignettes that self-assessments, can assume *K* integer values non-negative.

The model includes a self-assessment component and a component related to the vignettes.

#### Self-Evaluation Component

Assume that the actual response provided by the respondent *i-th* to the self-evaluation question is a latent one-dimensional random variable *µ_i_*, which is a linear function of a vector of parameters *β* and explanatory covariates *X_i_* which add up to a random effect *η_i_*:

$\begin{array}{c} \mu_{i}=X_{i}\beta +\eta_{i}\\ \eta_{i}∼N\left(\mu_{i},\omega^{2}\right) \end{array}$

The parameters indicated with *β,* as in a classic ordinal probit model, define the effect of the covariates on the concept under investigation measured on an ordinal scale.

The level perceived by the subject *i-th* respect to *µ_i_*, is normally distributed and is however a random component unobservable *Y_i_**:

$Y_{i}^{\text{∗}}∼N\left(\mu_{i},\sigma^{2}\right)$

The observation mechanism that allows to move from perceived level to the one observed, divides the latent variable *Y_i_** by means of the thresholds, as for the general ordinal probit model.

Yi=K  if τiK−1≤Zij∗≤τiK

The thresholds in this case, vary depending on the respondent for which the threshold levels are no longer constant but vary between subjects and are linear function of a vector of explanatory covariates *Vi* by γ parameters which define the effect of the subject characteristic on the question interpretation:

$\begin{array}{c} \tau_{i}^{1}=\gamma^{1}V_{i}\\ \tau_{i}^{K}=\tau_{i}^{K-1}+\gamma^{k}V_{i}\left(K=2,...,\;K-1\right) \end{array}$

Using only the component related to self-evaluation, it is not possible to separate the parameters β and γ, for that reason, we must use the information provided by the vignettes modeling also this component.

#### Vignettes Component

The model assumes that the respondent perceives the level of assessment on the vignette *j-th*, presented by the researcher, through a mechanism of observation defined on a latent random variable *Z*_ij_* such that:

$Z_{\mathrm{ij}}^{\text{ ∗ }}∼N\;\left(\theta_{j},\sigma_{j}^{2}\right)$

You can see that *θ*, which represents the level perceived for the vignettes, only varies depending on the *j-th* vignette and not according to the individual *i-th*, as is assumed in the model that each vignette is interpreted in the same way by the various respondents (*vignettes equivalence*).

Another assumption is that among individuals can vary the interpretation of the question pertaining to self-evaluation, but, each individual, in the same way, uses the measurement scale for answer to the the question pertaining to self-evaluation, both to evaluate the fictitious situations presented in the vignettes. This property is defined as *response consistency*.

The mechanism of discretization of continuous latent variable *Z*_ij_* is the same, whereas the number of values that can assume the score of a vignette *j-th* are *K* as for the question relative to self-evaluation:

Z∗ij=K if τiK−1≤Zij * ≤τiK

The thresholds are determined by the same linear combination expected in the case of modeling on self-assessment, or through the γ coefficients and explanatory covariates *Vi*:

$\begin{array}{c} \tau_{i}^{1}=\gamma^{1}V_{i}\\ \tau_{i}^{K}=\tau_{i}^{K-1}+\gamma^{k}V_{i}\;\left(K=2,...,\;K-1\right) \end{array}$

The cut points of the latent random variable are the same Z*ij and Yi*, and, unlike the ordinal probit model these vary according to the individual characteristics.
